# Millimeter-Wave Imaging for Idiopathic Scoliosis Screening: Diagnostic Accuracy Study

**DOI:** 10.2196/92125

**Published:** 2026-07-06

**Authors:** Wei Li, Chang Liu, Qinglin Zhang, Xiaotian Tong, Yu Xia, Zhenli Zhao, Cailiang Shen

**Affiliations:** 1 Department of Orthopedics and Spine Surgery First Affiliated Hospital of Anhui Medical University He Fei China; 2 Anhui Yuanshuo Terahertz Technology Co., Ltd He Fei China

**Keywords:** idiopathic scoliosis, early detection, millimeter-wave imaging, diagnostic accuracy, nonionizing radiation

## Abstract

**Background:**

Idiopathic scoliosis is a common 3D spinal deformity with a global prevalence of 2% to 3% in adolescents. Early detection is crucial for timely intervention and preventing curve progression. Although standing full-spine radiography with Cobb angle measurement remains the diagnostic gold standard, its time-consuming nature limits its utility for large-scale screening. Safe, rapid, and noninvasive screening methods are urgently needed to identify high-risk individuals while reserving x-rays for definitive diagnosis.

**Objective:**

This study aimed to evaluate the diagnostic accuracy of a custom-developed millimeter-wave imaging system in patients with suspected scoliosis, with radiographic Cobb angle measurement serving as the reference standard, and explore its potential as a first-line screening tool.

**Methods:**

This prospective diagnostic accuracy study enrolled consecutive outpatients with suspected scoliosis. All participants underwent rapid millimeter-wave imaging scanning (K_a_ band, 29-40 GHz with no undressing required) followed by standard standing full-spine radiography. Scoliosis was defined as a Cobb angle of 10° or higher. Millimeter-wave images were evaluated using four morphological indicators: (1) shoulder height asymmetry (≥2 cm), (2) trunk lateral shift (≥2 cm), (3) waistline contour asymmetry (≥5°), and (4) lower-limb height difference (≥1 cm). A multiparameter integration strategy classified screening as positive if more than 2 indicators exceeded threshold values. Cobb angle measurements were performed independently by 2 experienced orthopedic surgeons blinded to millimeter-wave image results and each other’s assessments. Diagnostic performance metrics (sensitivity, specificity, positive predictive value, negative predictive value, and overall accuracy) were calculated according to the STARD (Standards for Reporting of Diagnostic Accuracy Studies) guidelines. A multivariate logistic regression model incorporating the 4 morphological parameters was constructed for receiver operating characteristic curve analysis.

**Results:**

Ultimately, 132 participants were included. Radiographic evaluation confirmed 98 (74.2%) cases with scoliosis (Cobb angle≥10°) and 34 (25.8%) negative cases. Millimeter-wave imaging achieved an overall accuracy of 86.4% (114/132; 95% CI 76.5%-94.7%), sensitivity of 85.7% (84/98; 95% CI 75.1%-96.5%), specificity of 88.2% (30/34; 95% CI 70.7%-97.6%), positive predictive value of 95.5%, and negative predictive value of 68.2%. Receiver operating characteristic curve analysis of the multivariate logistic regression model exhibited an area under the curve of 0.862 (95% CI 0.802-0.922), with a sensitivity of 92.9% (91/98) and specificity of 38.2% (13/34). Notably, sensitivity was lower for mild curves (Cobb angle=10°-20°) than for moderate or severe curves (≥20°).

**Conclusions:**

Millimeter-wave imaging represents a feasible, rapid, and non-ionizing radiation screening method for scoliosis with good diagnostic accuracy (accuracy=114/132, 86.4%; sensitivity=84/98, 85.7%; specificity=30/34, 88.2%). Its capability to penetrate clothing and rapid scanning time (approximately 2 seconds) make it suitable for large-scale screening applications. As a first-line screening tool, this method identifies high-risk individuals for targeted referral and definitive radiography. It follows the “as low as reasonably achievable” principle, optimizing scoliosis screening and reducing unnecessary radiation exposure.

## Introduction

Idiopathic scoliosis is a common 3D spinal deformity characterized by coronal plane curvature, vertebral rotation, and alterations in the sagittal plane profile [1,2]. Among its various subtypes, adolescent idiopathic scoliosis constitutes the predominant form affecting children and adolescents, with an estimated global prevalence of 2% to 3% [3-6]. Early detection during the adolescent growth spurt is of paramount importance as timely intervention can delay or halt deformity progression, thereby reducing the risk of progression to severe curves requiring surgical correction [7,8].

Currently, standing full-spine radiography with Cobb angle measurement remains the gold standard for diagnosing and monitoring idiopathic scoliosis. According to guidelines from the Scoliosis Research Society and the International Society on Scoliosis Orthopaedic and Rehabilitation Treatment, scoliosis is defined as a coronal curvature of 10° or more as measured using the Cobb method [7]. Although radiography provides high diagnostic accuracy, repeated exposure to ionizing radiation raises concerns regarding cumulative cancer risk, particularly in pediatric patients who may require long-term follow-up [9,10]. Moreover, the procedure is relatively time-consuming, limiting its practicality for large-scale population screening [11].

Accordingly, there is a growing demand for safe, rapid, and noninvasive screening methods capable of identifying individuals at elevated risk of scoliosis while reserving radiography for confirmatory diagnosis [12]. Several radiation-free approaches, including surface topography [13] and optical scanning [14], have been investigated. However, these techniques are frequently constrained by their sensitivity to clothing and ambient lighting, as well as workflow inefficiencies that limit their applicability in high-throughput screening settings [15,16].

Millimeter-wave imaging has been widely adopted in security screening due to its ability to penetrate clothing and generate high-resolution images without ionizing radiation [17,18]. On the basis of frequency-modulated continuous wave radar [19] and synthetic aperture radar processing principles [20], this technology enables reconstruction of surface topography and subsurface contours. When applied to the human back, millimeter-wave imaging has the potential to capture clinically relevant morphological features associated with spinal deformity in a rapid, objective, and privacy-conscious manner (Multimedia Appendices 1 and 2).

Despite the theoretical advantages of millimeter-wave imaging technology, no studies have evaluated its clinical utility for scoliosis detection. The objective of this study was to evaluate the diagnostic accuracy of a custom-developed millimeter-wave imaging system for detecting scoliosis in patients with suspected spinal deformity using radiographic Cobb angle measurement as the reference standard. We hypothesized that millimeter-wave imaging would demonstrate high sensitivity and acceptable specificity, supporting its potential application as a first-line screening tool consistent with the “as low as reasonably achievable” principle.

## Methods

### Ethical Considerations

This prospective diagnostic accuracy study was conducted in accordance with the Declaration of Helsinki and approved by the institutional review board of the First Affiliated Hospital of Anhui Medical University (approval PJ 2025-16-05). Written informed consent was obtained from all participants and their legal guardians prior to enrollment.

### Study Population

The inclusion criteria were as follows: (1) no prior history of scoliosis diagnosis; (2) suspected scoliosis based on positive physical examination findings, parental concern, or referral from school or community screening programs; and (3) ability to maintain a standing posture during imaging examinations.

The exclusion criteria were as follows: (1) inability to tolerate standing examination due to severe orthostatic intolerance or balance disorders, (2) severe skin conditions or open wounds on the back that might affect imaging quality, or (3) any condition judged by the investigator as potentially compromising patient safety or data integrity.

### Millimeter-Wave Imaging System and Acquisition

The scoliosis screening system used a custom-developed millimeter-wave scanner operating in the K_a_ band (29-40 GHz). This frequency range was selected to balance penetration through common clothing materials with spatial resolution sufficient for detailed surface topography mapping [21]. The system integrated frequency-modulated continuous wave radar for precise range resolution [19] and synthetic aperture radar processing via the range migration algorithm [20,22] to generate high-resolution azimuthal imagery. The fusion of these techniques produced a detailed 3D scattering map of the back’s surface and underlying contours.

**Figure 1:**
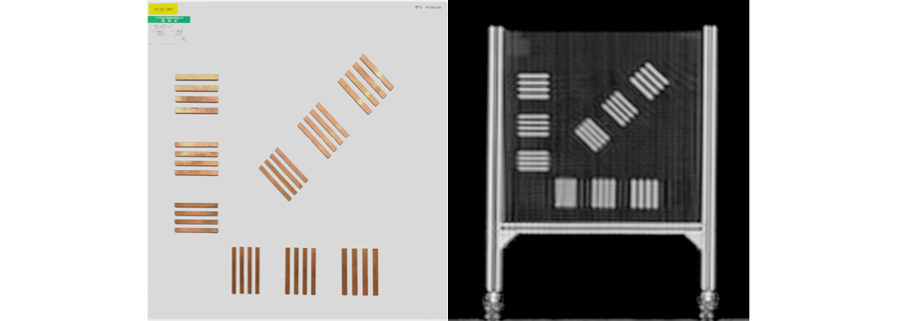
Image of the calibration test board and a scan of the board.

The reconstructed volume was characterized by a voxel matrix of 192 (width) × 448 (height) × 128 (depth) with spacings of 4.2 mm (horizontal), 3.6 mm (vertical), and 4.0 mm (depth). A maximum intensity projection algorithm was applied for initial visualization and qualitative analysis. The scanning procedure was completed within approximately 2 seconds, and calibration was verified using standardized test boards to ensure imaging accuracy. The image of the calibration test board is shown in Figure 1.

### Imaging Protocol

**Figure 2:**
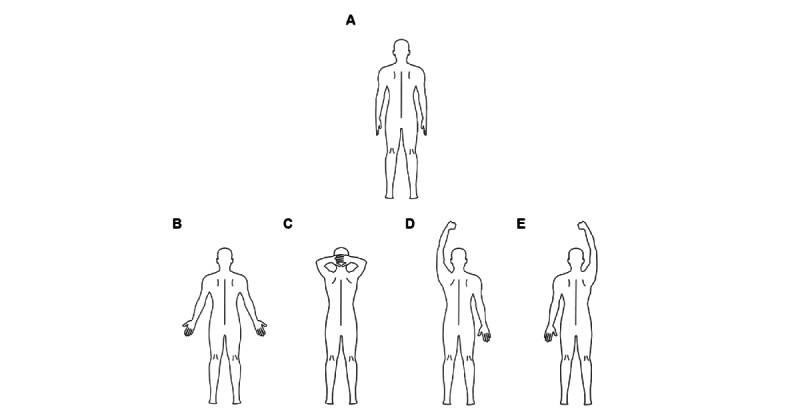
Schematic illustration of millimeter-wave image scanning postures.

Each participant underwent millimeter-wave scanning in 5 standardized standing positions (as illustrated in Figure 2): natural standing position (posture A), palms externally rotated (posture B), hands behind the head (posture C), and single-arm elevation (postures D and E).

Considering reproducibility and stability, this study used only the natural standing position (posture A) for quantitative analysis, with the remaining 4 postures reserved for qualitative assessment.

Participants were scanned while wearing lightweight clothing (including athletic wear, single-layer garments, or T-shirts) without the need for disrobing, thereby substantially preserving patient privacy and dignity. Metallic items (such as underwire bras) that could potentially interfere with imaging quality were removed when present (Multimedia Appendix 3).

All participants underwent standard standing anteroposterior and lateral full-spine radiography using standardized imaging parameters (85 kV; 20-55 mAs; focus-film distance: 180 cm). Regarding examination sequence, participants first underwent millimeter-wave scanning followed by standard radiographic examination as the definitive diagnostic reference. Both examinations were completed on the same day to minimize potential changes in spinal alignment.

The blinding protocol required millimeter-wave image analysts to be blinded to radiographic results and radiographic assessors to be blinded to millimeter-wave results. Cobb angle measurements were performed independently by 2 experienced orthopedic surgeons, with assessors blinded to each other’s results and to millimeter-wave findings. All image identifiers were removed prior to analysis, and an independent data management system was used to prevent unblinding.

### Image Analysis and Screening Criteria

Millimeter-wave images were analyzed using a predefined protocol focusing on 4 clinically relevant morphological indicators [23-29]: shoulder height asymmetry, trunk lateral shift, waistline contour asymmetry, and lower-limb height discrepancy.

#### Assessment of Shoulder Height Asymmetry

**Figure 3:**
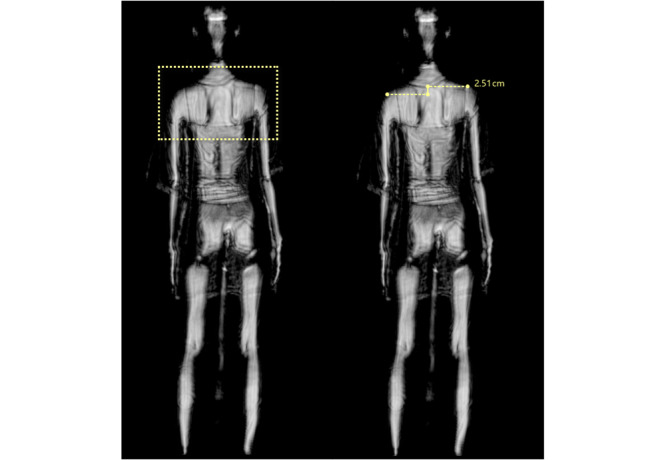
Assessment of shoulder balance.

Shoulder balance was evaluated by first interactively selecting the shoulder regions from the millimeter-wave maximum intensity projection image. A least squares fitting algorithm was applied to automatically identify the bilateral acromion points, with submillimeter precision in coordinate extraction. The vertical height difference (ΔH) was calculated as the absolute difference in the z-axis coordinates of the acromion points. The assessment criteria followed those by Lee et al [23] and other clinical standards [24], where ΔH of less than 2 cm was classified as normal and ΔH of 2 cm or more indicated abnormal shoulder imbalance (as illustrated in Figure 3).

#### Assessment of Trunk Lateral Shift

**Figure 4:**
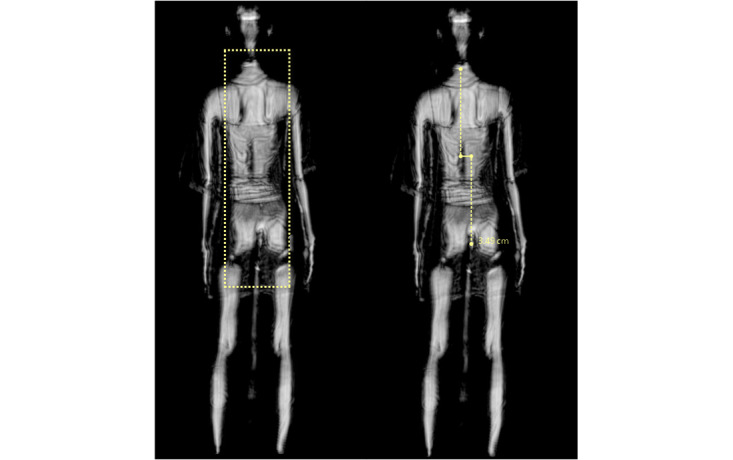
Assessment of trunk lateral shift.

Trunk alignment was quantified by measuring the horizontal offset distance (ΔL) between the midpoint of the cervicothoracic junction (C7-T1) and the gluteal cleft. In this study, the assessment of trunk lateral shift was performed by measuring the horizontal distance difference (ΔL) between the medial shoulder midpoint and the gluteal midline point. Following the work by Trobisch et al [25] and related studies on trunk balance [26,27], ΔL of less than 2 cm was classified as normal, and ΔL of 2 cm or more was classified as abnormal (as illustrated in Figure 4).

#### Assessment of Waistline Contour Asymmetry

**Figure 5:**
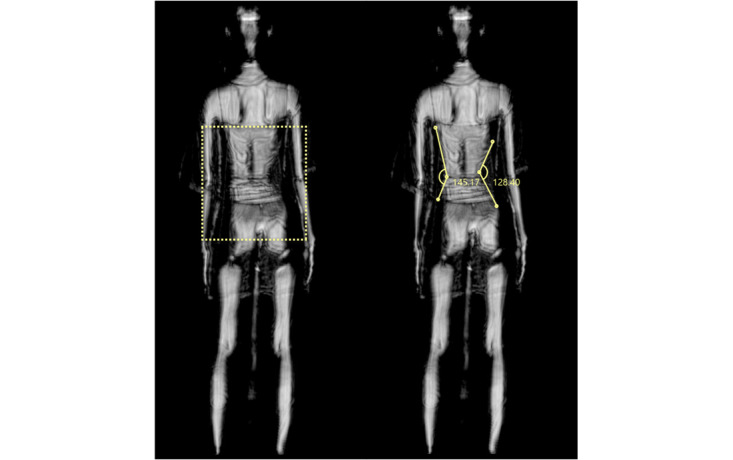
Assessment of waistline contour asymmetry.

Waistline symmetry was analyzed by delineating bilateral waistline contours through interactive annotation. Bezier curve fitting technology was used to extract curvature characteristics, and the tangent angle difference (ΔD) at the points of maximum curvature was calculated. Following the work by Qiu et al [28], ΔD of less than 5° was classified as normal, and ΔD of 5° or more indicated abnormal asymmetry (as illustrated in Figure 5).

#### Assessment of Lower-Limb Height Asymmetry

**Figure 6:**
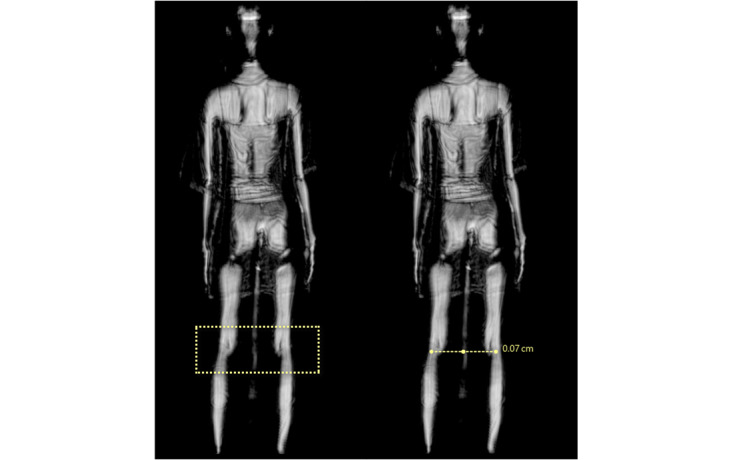
Assessment of lower-limb asymmetry.

Lower-limb discrepancy was assessed by interactively annotating the popliteal regions in the millimeter-wave images. The system automatically identified the highest points of the bilateral popliteal fossae with knee extension to standardize measurements. The height difference (ΔH) was computed. Following established clinical criteria [29], a threshold of ΔH of less than 1 cm defined the normal range, whereas ΔH of 1 cm or more indicated abnormality (as illustrated in Figure 6).

#### Judgment Criteria

On the basis of a multiparameter integrated strategy, a scan was categorized as positive for scoliosis screening if 2 or more of the 4 morphological indicators met or exceeded their predefined abnormal thresholds; otherwise, it was classified as negative (Multimedia Appendix 4).

### Statistical Analysis

Diagnostic performance was assessed using sensitivity, specificity, positive predictive value, negative predictive value, and overall accuracy in accordance with the STARD (Standards for Reporting of Diagnostic Accuracy Studies) guidelines [30]. Continuous variables with normal distribution were expressed as means and SDs and compared between groups using the independent-sample *t* test (2-tailed), whereas nonnormally distributed data were analyzed using the Mann-Whitney *U* test. All statistical analyses were performed using SPSS (version 22.0; IBM Corp), with statistical significance defined as a 2-tailed *P* value of less than .05.

## Results

### Patient Characteristics and Group Distribution

Between July 2024 and December 2025, a total of 165 consecutive patients presenting to the spinal surgery outpatient clinic with suspected scoliosis were screened for eligibility. Of these 165 patients, 19 (11.5%) were excluded for failing to meet the inclusion criteria (eg, not first-time presentation). Of the remaining 146 patients, 14 (9.6%) were subsequently excluded based on the exclusion criteria: 9 (64.3%) for inability to undergo radiographic examination (patient refusal or contraindications) and 5 (35.7%) for excessive perspiration on the back compromising image quality. Ultimately, 132 patients were enrolled and completed the study protocol, constituting the final analysis cohort.

Of the 132 patients enrolled in this study, 98 (74.2%) were confirmed to have scoliosis (Cobb angle≥10°) based on x-ray examination, whereas the remaining 34 (25.8%) had measurements of less than 10° and were classified as the nonscoliotic group. The baseline characteristics of the 2 groups are summarized in Table 1. There were no significant differences in age, height, or weight between the 2 groups (*P*>.05), ensuring the comparability of the study cohorts.

**Table 1:** Baseline characteristics of the study participants.

Characteristic			Scoliosis group (n=98)		Nonscoliosis group (n=34)		*P* value	
Age (y), mean (SD; range)			15.9 (9.3; 6-23)		14.7 (8.4; 6-22)		.22	
**Gender, n (%)**								.04
	Male	32 (32.7)		18 (52.9)				
	Female	66 (67.3)		16 (47.1)				
Height (cm), mean (SD; range)			157.2 (9.3; 122.3-184.4)		155.9 (8.4; 120.2-174.3)		.52	
Weight (kg), mean (SD; range)			49.1 (7.2; 26.2-78.9)		47.2 (5.8; 26.1-72.1)		.19	
Cobb angle (°), mean (SD; range)			28.5 (12.3; 10.5-87.5)		6.2 (2.1; 1.9-9.7)		<.001	

### Diagnostic Performance of Millimeter-Wave Imaging

The comparative analysis between millimeter-wave assessments and the x-ray reference standard yielded the results shown in Table 2. The overall diagnostic accuracy of the millimeter-wave imaging protocol was 86.4% (114/132). The technology demonstrated a high sensitivity of 85.7% (84/98) and a specificity of 88.2% (30/34). The positive predictive value was 95.5%, and the negative predictive value was 68.2%.

**Table 2:** Diagnostic performance of millimeter-wave imaging.

Parameter	Value (%; 95% CI)
Accuracy	86.4 (76.5-94.7)
Sensitivity	85.7 (75.1-96.5)
Specificity	88.2 (70.7-97.6)
PPV^a^	95.5 (94.5-96.7)
NPV^b^	68.2 (66.7-72.7)

^a^PPV: positive predictive value.

^b^NPV: negative predictive value.

### Analysis of Millimeter-Wave Morphological Parameters

With the exception of lower-limb length discrepancy, the remaining 3 morphological parameters demonstrated higher abnormality rates in the scoliosis group than in the nonscoliosis group as detailed in Table 3. Notably, shoulder height asymmetry and waistline contour asymmetry also exhibited relatively elevated abnormality rates in the nonscoliosis group (13/34, 38.2% and 11/34, 32.4%, respectively).

**Table 3:** Millimeter-wave imaging morphological parameter analysis.

Parameter	Abnormalities in the scoliosis group (n=98), n (%)	Abnormalities in the nonscoliosis group (n=34), n (%)
Shoulder height asymmetry	75 (76.5)	13 (38.2)
Trunk lateral shift	38 (38.8)	2 (5.9)
Waistline contour asymmetry	78 (79.6)	11 (32.4)
Lower-limb discrepancy	6 (6.1)	1 (2.9)

### Multivariate Model Analysis

**Figure 7:**
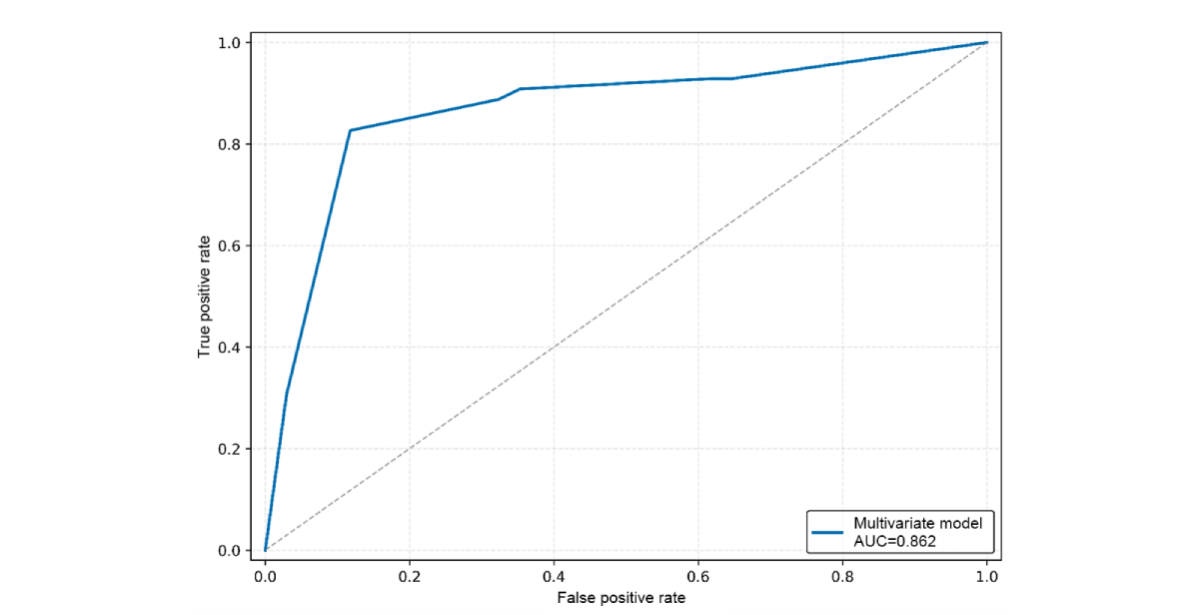
Receiver operating characteristic curve of the multivariate logistic regression model. AUC: area under the curve.

To further evaluate the integrated diagnostic capability of millimeter-wave imaging, a multivariate logistic regression model was developed incorporating the 4 morphological parameters. The receiver operating characteristic curve analysis of this composite model demonstrated superior performance compared to the individual parameters. The multivariate model achieved an area under the curve of 0.862 (95% CI 0.802-0.922), indicating excellent discriminatory ability (as illustrated in Figure 7).

**Figure 8:**
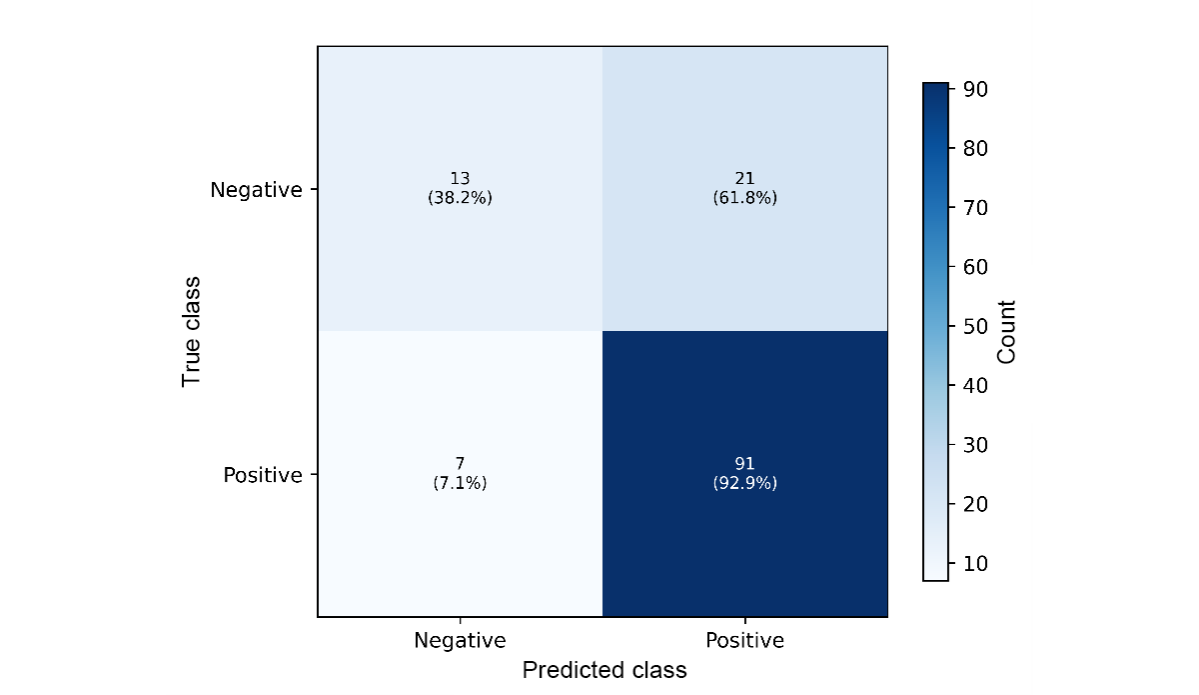
Confusion matrix detailing the performance of the multivariate model.

The detailed performance metrics of the model are shown in the confusion matrix (Figure 8). The model exhibited a sensitivity of 92.9% (91/98 true positives) and a specificity of 38.2% (13/34 true negatives), with an overall accuracy of 78.8% (104/132). This indicates that the methodology aligns with the primary goal of screening protocols, which is to prioritize sensitivity to avoid missed cases, thereby underscoring its value in clinical practice.

### Subgroup Analysis by Curve Severity

In a subgroup analysis of the 98 patients with scoliosis, the millimeter-wave imaging system demonstrated varying performance across different curve severity groups. The technology demonstrated high diagnostic accuracy in the moderate curve group (Cobb angle=20°-40°) and severe group (≥40°) compared to 75.4% (43/57) in the mild group (10°-20°).

## Discussion

### Principal Findings

This diagnostic accuracy study evaluated a custom-developed millimeter-wave imaging system in 132 pediatric outpatients with suspected scoliosis. The system demonstrated a sensitivity of 85.7% (84/98), specificity of 88.2% (30/34), and overall accuracy of 86.4% (114/132) for detecting scoliosis (Cobb angle≥10°). Each scan was completed in approximately 2 seconds, effectively minimizing motion artifacts. Notably, sensitivity was lower for mild curves (Cobb angle=10°-20°) than for moderate or severe curves.

As a non-ionizing modality, millimeter-wave imaging aligns with the “as low as reasonably achievable” principle and may reduce unnecessary radiation exposure in pediatric populations. The rapid acquisition time (approximately 2 seconds) and ability to scan through light clothing balanced efficiency with privacy preservation, supporting application in large-scale, high-throughput settings such as schools or communities. Moreover, the 3D surface data may enable future assessment of parameters such as vertebral rotation, offering morphological information beyond that provided by conventional 2D radiography.

In summary, this technology is suitable for initial scoliosis screening. Key advantages include (1) absence of ionizing radiation, avoiding cumulative cancer risks from repeated radiographic examinations and supporting longitudinal monitoring; (2) ability to image through everyday clothing, preserving patient dignity and addressing privacy concerns relevant to adolescents; (3) rapid scanning, facilitating efficient large-scale screening; and (4) 3D morphological data that complement planar radiographic findings. Current scoliosis screening techniques based on angular measurement primarily include the use of a scoliometer for assessing the angle of trunk rotation (ATR) and optical surface topography systems. Reported sensitivities for these methods typically range from approximately 70% to 86% [31,32]. In comparison, the millimeter-wave imaging system used in this study achieved a sensitivity of 85.7% (84/98), which is comparable to the performance of existing non–radiation‑based screening technologies. The device can be positioned as a triage tool to identify high-risk individuals requiring confirmatory radiographic evaluation. It should be emphasized that this system does not replace Cobb angle measurement, and definitive diagnosis still requires standard radiography.

### Limitations

This study has several limitations that should be acknowledged. First, diagnostic accuracy was significantly lower in the mild scoliosis group (Cobb angle=10°-20°) than in the moderate (20°-40°) and severe (≥40°) groups. This difference arose because, under the current diagnostic criteria, some imaging-confirmed mild scoliosis cases were classified as negative during initial screening when at least one quantitative parameter did not meet the predefined threshold, resulting in false-negative outcomes (Multimedia Appendix 5). Moreover, although multi-posture analysis often identifies compensatory asymmetries indicative of scoliosis (eg, scapular asymmetry), these features are not fully captured in single-posture or single-parameter assessments. To preserve the objectivity of this blinded diagnostic accuracy study, qualitative findings from multi-posture observations were not incorporated into the formal statistical analysis, nor were they used to retrospectively revise the initial binary (positive or negative) classification based on the predefined quantitative thresholds.

Second, this was a diagnostic accuracy study conducted in patients with suspected scoliosis referred for clinical evaluation, not a screening study in an asymptomatic population. Future multicenter studies with larger and more diverse populations are needed to further validate the clinical applicability of this technology.

Third, we did not include ATR measurements for direct comparison with existing clinical screening tools. In addition, we did not perform direct comparison between clothed and unclothed measurements or between millimeter-wave imaging and optical surface topography systems. Future studies should incorporate direct comparison among ATR measurements, optical surface topography systems, and millimeter-wave imaging.

Finally, diagnostic accuracy was lower for mild scoliosis (10°-20°) as subtle asymmetries may not always meet quantitative threshold criteria. Future integration of machine learning algorithms for automated analysis may improve detection of mild curves [32-35].

### Conclusions

Millimeter-wave imaging represents a promising advancement in scoliosis screening. It provides a rapid, safe, and privacy-preserving approach, achieving a diagnostic performance comparable to initial clinical assessments while offering greater objectivity. As an effective preliminary screening step prior to radiographic evaluation, this technology has the potential to optimize health care resource use. Overall, it shows substantial promise for enabling large-scale, routine scoliosis screening in a safe and practical manner, consistent with recommendations from leading clinical organizations.

## Appendix

Multimedia Appendix 1 Scanning image method.

Multimedia Appendix 2 Scanning working principle.

Multimedia Appendix 3 Scan workflow demonstration.

Multimedia Appendix 4 Data measure.

Multimedia Appendix 5 Reinterpretation of false-negative cases through multi-postural millimeter-wave imaging.

## Abbreviations

ATR: angle of trunk rotation
STARD: Standards for Reporting of Diagnostic Accuracy Studies

## Data Availability

The data used to support the findings of this study are available from the corresponding author on reasonable request.
