# Supplementary material for: Millimeter-Wave Imaging for Idiopathic Scoliosis Screening: Diagnostic Accuracy Study
Source: JMIR Pediatr Parent. 2026 Jul 6;9:e92125. doi: 10.2196/92125 (PMC13386118; doi:10.2196/92125)
Supplement: Multimedia Appendix 5 [file pediatrics_v9i1e92125_app5.docx]

**Reinterpretation of False-Negative Cases through Multi-Postural Millimeter-Wave Imaging**

Under the current diagnostic criteria, a subset of radiographically confirmed scoliosis cases (Cobb angle ≥10°) were initially classified as negative based on individual parameter thresholds, representing false-negative assessments. However, the millimeter-wave imaging protocol acquired five distinct postural views per subject, enabling a comprehensive evaluation of spinal morphology. The multi-postural analysis frequently revealed compensatory asymmetries indicative of scoliosis—such as scapular asymmetry—that were not fully captured by single-posture or single-parameter assessments.

**Case-Based Analysis:**

Case 1 presented a Cobb angle of 13.5°, meeting the radiographic criterion for scoliosis. While trunk lateral shift (0.3 cm) and waistline contour asymmetry (1.08°) fell within normal limits, significant shoulder height asymmetry (2.91 cm) was observed. However, features consistent with scapular asymmetry were evident in postures d and e, and waistline asymmetry became pronounced in posture c.

Case 2, with a Cobb angle of 16.5°, exhibited marked waistline contour asymmetry (10.03°) but normal shoulder height asymmetry (1.37 cm) and trunk lateral shift. This case also indicates that human body symmetry varies significantly under different postures, thus can serve as a positive indicator in assessment.

These observations suggest that the multi-postural imaging approach enhances detection sensitivity by capturing dynamic morphological changes not apparent in a single static pose. It is important to note that, to maintain the objectivity of the blinded diagnostic accuracy study, these qualitative multi-postural observations were not used to retrospectively alter the initial binary classification (positive/negative) based on the predefined quantitative thresholds during the formal statistical analysis.

**Case1: Index 17**

| 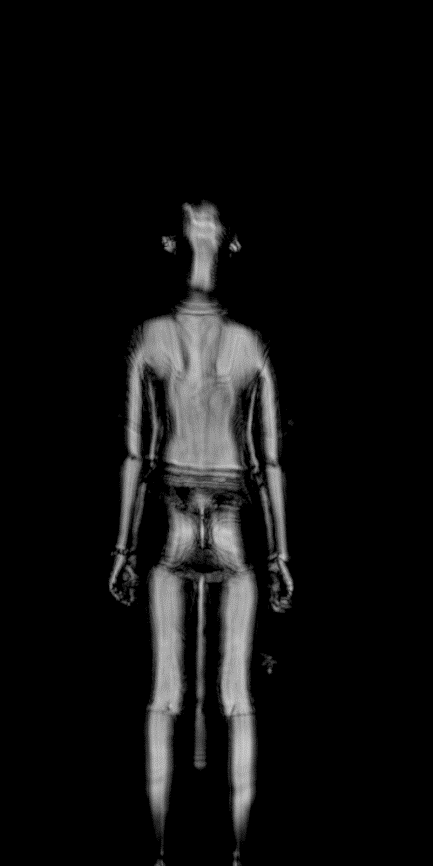 | 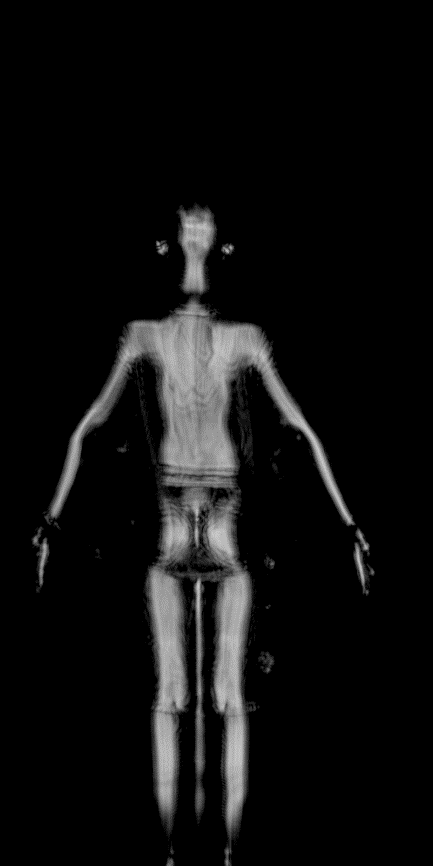 | | 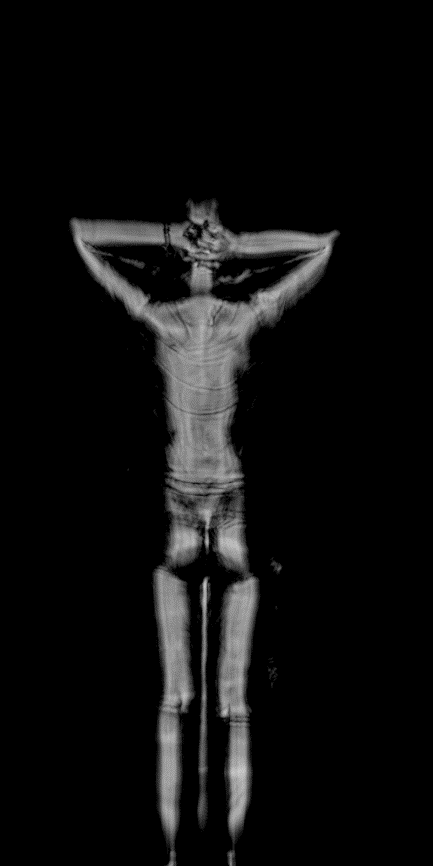 |
| --- | --- | --- | --- |
| 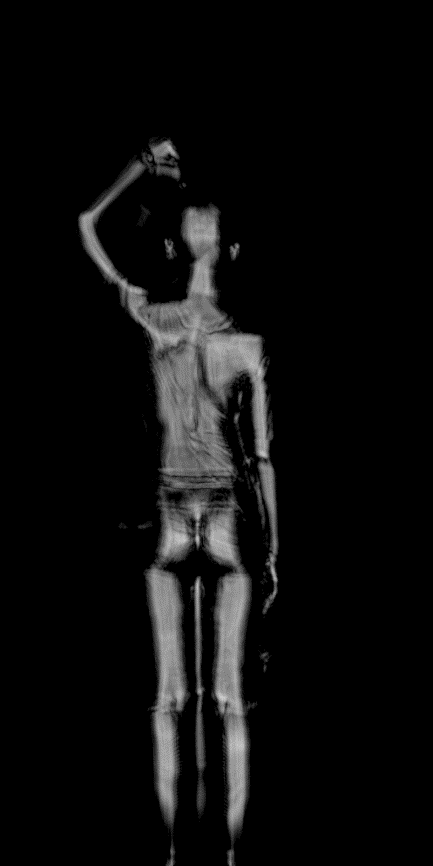 | 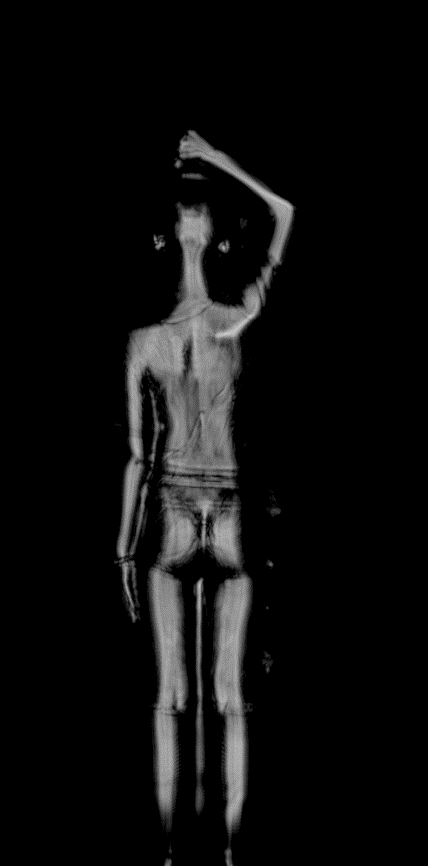 | | 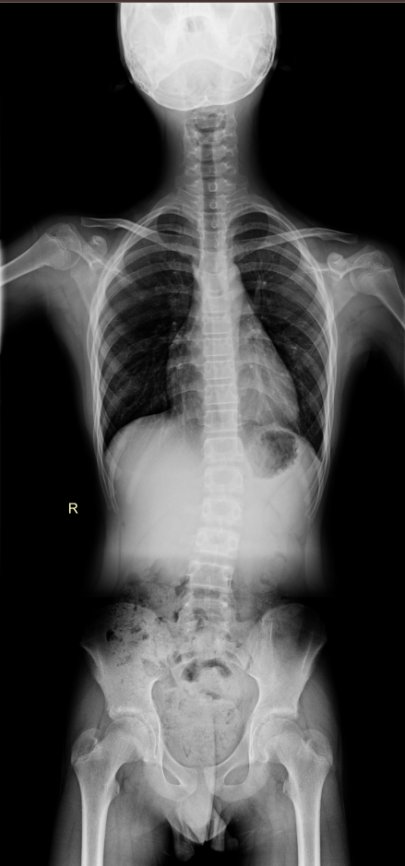 |
| X Ray Cobb Angle | | 13.5 | |
| Shoulder Height Asymmetry (ΔH,cm) | | 2.91 | |
| Trunk Lateral Shift (ΔL,cm) | | 0.3 | |
| Waistline Contour Asymmetry (ΔD，°) | | 1.08 | |
| Shoulder Height Asymmetry (ΔH,cm) | | 0.54 | |

**Case2: Index 73**

| 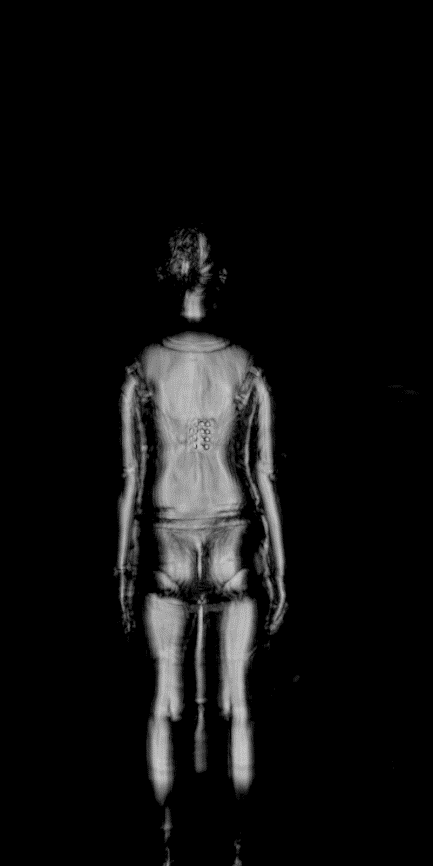 | 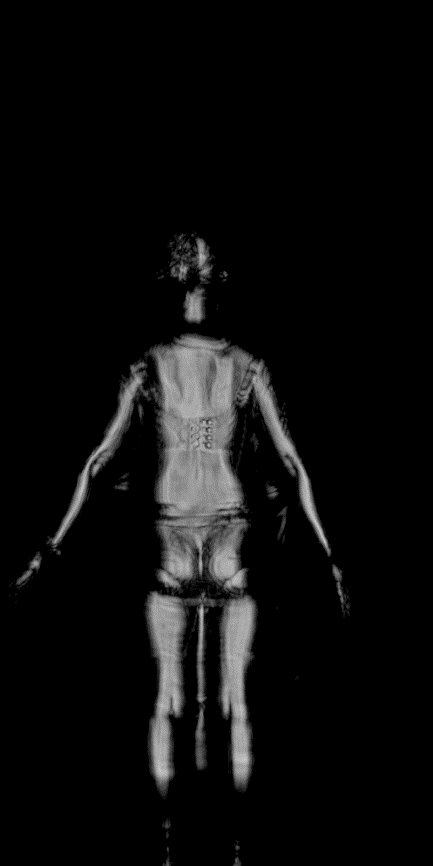 | | 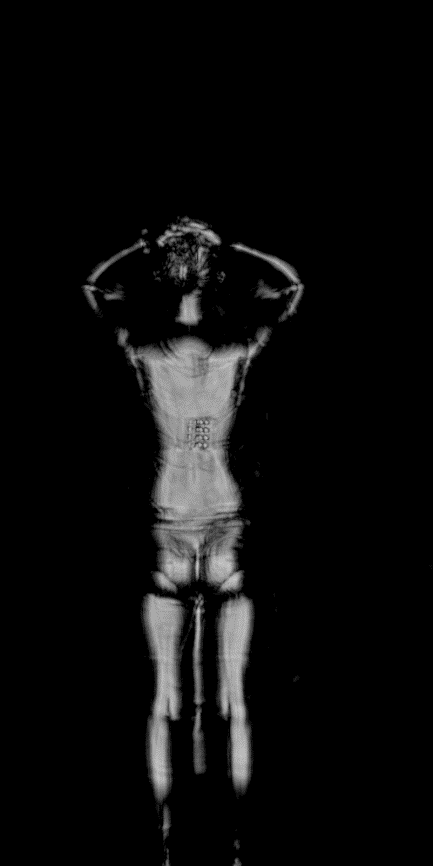 |
| --- | --- | --- | --- |
| 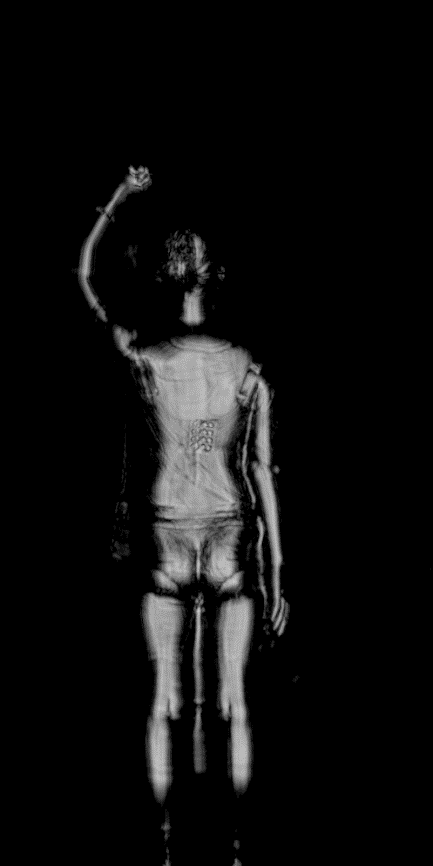 | 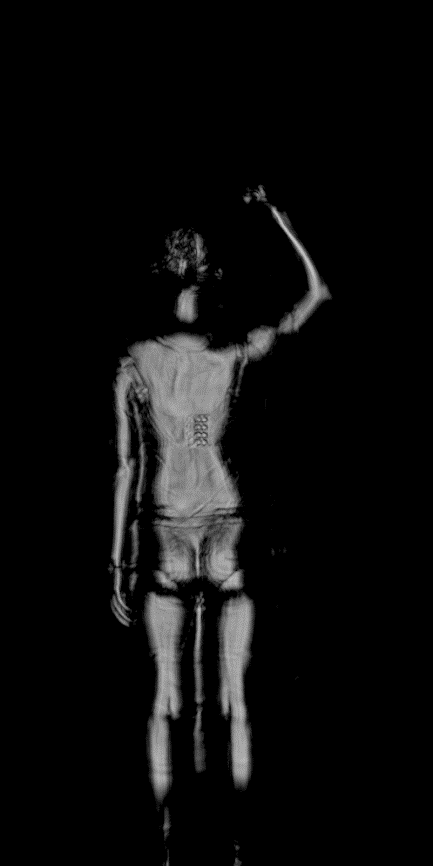 | | 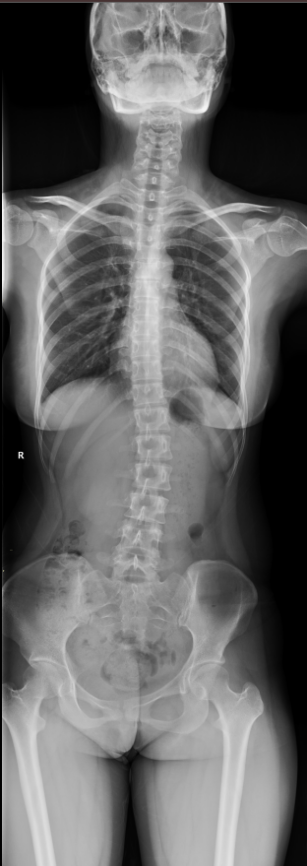 |
| X Ray Cobb Angle | | 16.5 | |
| Shoulder Height Asymmetry (ΔH,cm) | | 1.37 | |
| Trunk Lateral Shift (ΔL,cm) | | 1.43 | |
| Waistline Contour Asymmetry (ΔD，°) | | 10.03 | |
| Shoulder Height Asymmetry (ΔH,cm) | | 0.07 | |
